# Identification of DNA-Repair-Related Five-Gene Signature to Predict Prognosis in Patients with Esophageal Cancer

**DOI:** 10.3389/pore.2021.596899

**Published:** 2021-03-30

**Authors:** Lin Wang, Xueping Li, Lan Zhao, Longyang Jiang, Xinyue Song, Aoshuang Qi, Ting Chen, Mingyi Ju, Baohui Hu, Minjie Wei, Miao He, Lin Zhao

**Affiliations:** ^1^ Department of Pharmacology, School of Pharmacy, China Medical University, Shenyang, China; ^2^ Liaoning Key Laboratory of Molecular Targeted Anti-tumor Drug Development and Evaluation, Liaoning Cancer Immune Peptide Drug Engineering Technology Research Center, Key Laboratory of Precision Diagnosis and Treatment of Gastrointestinal Tumors, Ministry of Education, China Medical University, Shenyang, China

**Keywords:** prognostic biomarkers, DNA repair, overall survival, esophageal cancer, small molecular drugs, targeted therapy

## Abstract

Esophageal cancer (ESCA) is a leading cause of cancer-related mortality, with poor prognosis worldwide. DNA damage repair is one of the hallmarks of cancer. Loss of genomic integrity owing to inactivation of DNA repair genes can increase the risk of cancer progression and lead to poor prognosis. We aimed to identify a novel gene signature related to DNA repair to predict the prognosis of ESCA patients. Based on gene expression profiles of ESCA patients from The Cancer Genome Atlas and gene set enrichment analysis, 102 genes related to DNA repair were identified as candidates. After stepwise Cox regression analysis, we established a five-gene prognostic model comprising DGCR8, POM121, TAF9, UPF3B, and BCAP31. Kaplan-Meier survival analysis confirmed a strong correlation between the prognostic model and survival. Moreover, we verified the clinical value of the prognostic signature under the influence of different clinical parameters. We found that small-molecule drugs (trametinib, selumetinib, and refametinib) could help to improve patient survival. In summary, our study provides a novel and promising prognostic signature based on DNA-repair-related genes to predict survival of patients with ESCA. Systematic data mining provides a theoretical basis for further exploring the molecular pathogenesis of ESCA and identifying therapeutic targets.

## Introduction

Esophageal cancer (ESCA) is the sixth leading cause of cancer-related deaths worldwide, and its mortality has continued to increase [1]. ESCA has a poor prognosis due to early metastasis, and a 5-years overall survival (OS) rate is around 15% [2, 3]. Even in the same cancer stage of ESCA patients, patient prognosis may be different. Therefore, it is imperative to construct prognostic biomarkers that can be used to judge the survival outcomes of patients with ESCA. Clinical oncologists can also use these markers to determine whether adjuvant treatment is needed. Owing to various genetic and phenotypic alterations that have been reported in ESCA, gene biomarkers have gradually become a cost-effective and precise method for predicting the prognosis of ESCA patients [4]. However, polymorphisms of genes and tumor heterogeneity mean that single-gene biomarkers are inadequate [5]. Thus, the search for prognostic markers in cancer patients has increasingly focused on multi-gene biomarkers [6].

Gene expression analysis can provide a means of identifying potential prognostic markers related to survival. In recent years, many studies have shown that various gene changes precede deterioration in prognosis in ESCA patients. Importantly, it has been reported that genomic DNA is highly susceptible to damage and can be influenced by different types of chemotherapy drugs. The genomic instability induced by DNA damage can result in cell apoptosis and tumorigenesis. The DNA repair process is often blocked or destroyed in cancer cells, enabling them to rapidly evolve and adapt, which ultimately drives the development of cancer lesions and metastasis [7]. In addition, defective DNA repair genes can promote cell aging, apoptosis and proliferation, make carriers prone to cancer [8], and change the sensitivity of cancers to chemotherapy. Therefore, DNA damage repair, as one of the hallmarks of cancer, is indispensable for maintaining the genomic integrity of the cell. Recent studies have identified single biomarkers related to DNA repair in ESCA or its subtypes that could predict patients’ prognosis [9–11]. However, there is limited evidence regarding combined biomarkers of genes related to DNA repair in ESCA. Therefore, there is an urgent need to construct a prognostic gene signature based on DNA repair pathways for use in patients with ESCA.

The Cancer Genome Atlas (TCGA) is an authoritative, large-scale collaborative work led by the National Cancer Institute and the National Human Genome Institute [12]. It can be used to analyze genomic and epigenetic changes in 33 human cancers at the DNA, RNA, protein, and epigenetic levels, thus supporting new discoveries and accelerating research progress to improve cancer diagnosis, treatment, and prevention [13]. TCGA provides a valuable resource for the cancer research community. It collects a large number of human cancer samples and normal tissues, enabling researchers to identify important genomic changes that may have key roles in the development of cancer, and facilitates deeper and broader research of the cancer genome [14]. Here, we analyzed ESCA data in TCGA to find reliable prognostic markers, and randomly divided the entire TCGA dataset into two groups for supplementary verification.

Based on TCGA data mining, we selected five genes (DGCR8, POM121, TAF9, UPF3B, and BCAP31) associated with DNA repair to construct a prognostic signature, and showed that this signature performed well in predicting the prognosis of patients. The results of the high-throughput data mining showed that our prognostic model could independently predict ESCA patients’ survival. The results also provide a theoretical basis for further exploring the molecular pathogenesis of ESCA and identifying therapeutic targets.

## Materials and Methods

### Data Acquisition and Pre-Processing

TCGA (https://cancergenome.nih.gov/, data release v23.0), a publicly available database, can be used for genomic analyses of 33 cancers (tumor samples and normal samples). We downloaded RNA expression data (fragments per kilobase million, FPKM) of 171 samples from the TCGA data portal. FPKM is a normalized estimation based on RNA sequencing data. The final expression levels of the FPKM data were determined by quantile normalization and log_2_ transformation using the “limma” R package. We also downloaded clinical information for all samples. We removed one sample owing to incomplete clinical information, leaving 170 samples (159 tumor samples and 11 normal samples) for further analysis. The clinical information included patients’ general characteristics (age, gender, and race), subtype of ESCA, survival status, pathologic stage (TNM), neoplasm status, tumor location, neoplasm histological grade, residual tumor status and others (Table 1). We also downloaded an independent dataset (accession number GSE38129; *n* = 60, 30 normal and 30 tumor) from the Gene Expression Omnibus database (https://www.ncbi.nlm.nih.gov/geo/) for external validation. The platform of this dataset was GPL571. These data were normalized by robust multi-array average to validate the results.

**TABLE 1:** Summary of clinical characteristics of ESCA patients in three cohorts.

Characteristic	Patients in entire TCGA set (*n* = 159), *n* (%)	Patients in subgroup 1 (*n* = 79), *n* (%)	Patients in subgroup 2 (*n* = 80), *n* (%)
Age (years)			
≤60	81 (50.94%)	44 (55.70%)	37 (46.25%)
>60	78 (49.06%)	35 (44.30%)	43 (53.75%)
Gender			
Female	23 (14.47%)	13 (16.46%)	10 (12.50%)
Male	136 (85.53%)	66 (83.54%)	70 (87.5%)
Histological type			
Esophagus adenocarcinoma, NOS	79 (49.69%)	36 (45.57%)	43 (53.75%)
Esophagus squamous cell carcinoma	80 (50.31%)	43 (54.43%)	37 (46.25%)
Vital status			
Alive	96 (60.38%)	47 (59.49%)	49 (61.25%)
Dead	63 (39.62%)	32 (40.51%)	31 (38.75%)
Pathologic stage			
Stage I-II	87 (54.72%)	46 (58.23%)	41 (51.25%)
Stage III-IV	68 (42.77%)	32 (40.51%)	36 (45.00%)
NA	4 (2.51%)	1 (1.26%)	3 (3.75%)
Race			
Asian	38 (23.90%)	22 (27.85%)	16 (20.00%)
Black or african american	5 (3.14%)	2 (2.53%)	3 (3.75%)
White	98 (61.64%)	50 (63.29%)	48 (60.00%)
NA	18 (11.32%)	5 (6.33%)	13 (16.25%)
N Classification			
N0-N1	133 (83.65%)	65 (82.28%)	68 (85.00%)
N2-N3	14 (8.80%)	7 (8.86%)	7 (8.75%)
NA	12 (7.55%)	7 (8.86%)	5 (6.25%)
T classification			
T1	25 (15.72%)	14 (17.72%)	11 (13.75%)
T2-T4	132 (83.02%)	63 (79.75%)	69 (86.25%)
NA	2 (1.26%)	2 (2.53%)	0 (0.00%)
M classification			
M0	126 (79.25%)	59 (74.68%)	67 (83.75%)
M1	15 (9.43%)	7 (8.86%)	8 (10.00%)
NA	18 (11.32%)	13 (16.46%)	5 (6.25%)
Neoplasm cancer status			
Tumor free	91 (57.23%)	49 (62.03%)	42 (52.50%)
With tumor	58 (36.48%)	26 (32.91%)	32 (40.00%)
NA	10 (6.29%)	4 (5.06%)	6 (7.5%)
Tumor central location			
Distal	111 (69.81%)	54 (68.35%)	57 (71.25%)
Mid	41 (25.79%)	22 (27.85%)	19 (23.75%)
Proximal	6 (3.77%)	3 (3.80%)	3 (3.75%)
NA	1 (0.63%)	0 (0.00%)	1 (1.25%)
Neoplasm histologic grade			
G1	16 (10.06%)	6 (7.6%)	10 (12.5%)
G2	65 (40.88%)	33 (41.77%)	32 (40.00%)
G3	43 (27.05%)	23 (29.11%)	20 (25.00%)
NA	35 (22.01%)	17 (21.52%)	18 (22.50%)
Residual tumor			
R0	119 (74.84%)	60 (75.95%)	59 (73.75%)
R1+R2	13 (8.18%)	3 (3.80%)	10 (12.50%)
NA	27 (16.98%)	16 (20.25%)	11 (13.75%)
Lymph node metastasis			
NO	83 (52.20%)	34 (43.04%)	49 (61.25%)
Yes	43 (27.04%)	27 (34.18%)	16 (20.00%)
NA	33 (20.76%)	18 (22.78%)	15 (18.75%)

Abbreviations: ESCA, esophageal cancer; NA, not available.

### Screening DNA-Repair-Related Genes by Gene Set Enrichment Analysis

The gene set enrichment analysis (GSEA, http://www.broadinstitute.org/gsea/index.jsp) included 1320 gene sets and showed its distinction in gene detection by testing gene sets but not individual gene. It was determined whether a given gene pathway shows statistically significant differences between a cancer group and a normal group [15, 16]. Here, we used GSEA to identify significant differences in DNA repair pathways between the ESCA group and the normal group, using gene expression profile data for ESCA. We also obtained 102 DNA-repair-related genes as candidates for further analysis.

For deeper analysis, we constructed a protein-protein interaction network for these 102 genes using Metascape (http://metascape.org) [17], which provides biological pathways obtained through independent and orthogonal experiments on datasets of more than 40 knowledgebase. *p* < 0.05 is generally considered to represent significantly enriched pathways. Using molecular complex detection (MCODE), it can identify closely related protein groups, with biological function annotations for each group. We then explored the relationships between the 102 DNA-repair-related genes and biological pathways using gene ontology (GO) and Kyoto Encyclopedia of Genes and Genomes (KEGG) enrichment analysis with Metascape.

### Identification of DNA-Repair-Related Genes and Construction of Prognostic Model

In order to identify survival-related genes in DNA repair gene sets, univariate Cox linear proportional hazard regression (PHR) analysis was performed with the “univariate” R package. Furthermore, in order to identify independent prognostic factors and construct a prognostic model, we performed multivariate Cox linear PHR analysis with the “multivariate” R package. Finally, we constructed a prognostic signature comprising five genes that could predict the prognosis of ESCA patients. Based on gene expression values and regression coefficients, we developed a risk scoring system to predict the survival of patients. The equation is as follows

$$\text{Risk score}=\sum_{i}^{n}\text{Exp}i\;*\;\beta i$$

where Exp represents the gene expression level, and *β* is the partial regression coefficient of independent variables for each gene. We ranked the patients into two groups (high and low risk) using the median risk value.

Furthermore, we performed deeper analysis of the five genes using GeneMANIA (http://www.genemania.org), which can identify functionally similar genes using a wealth of genomics and proteomics data and indicate the function of these genes [18]. We uploaded the selected genes to GeneMANIA to identify interacting genes and analyze gene functions. Mutational analysis was carried out, and the drug sensitivities and biological functions of the five genes were examined using GSCALite (http://bioinfo.life.hust.edu.cn/web/GSCALite/) [19], which is widely used for gene set analysis in various cancers. The structures of potential drug molecules were visualized using PubChem (https://pubchem.ncbi.nlm.nih.gov/). Alterations of the five genes in ESCA were shown with cBioPortal (http://www.cbioportal.org/).

### Validation of Five-Gene Prognostic Signature in ESCA Patients

The entire dataset of TCGA patients with ESCA (*n* = 159) were randomly separated into two subgroups, denoted TCGA subgroup 1 (*n* = 79; Table 1) and TCGA subgroup 2 (*n* = 80; Table 1). The prognostic signature was identified in the entire TCGA dataset and validated in all three groups (the TCGA entire group and the two subgroups). Using the risk score formula, we calculated the risk value for each patient, and divided patients into two (high and low) groups by the median value. In order to validate the predictive capability of the prognostic signature, Kaplan-Meier (K-M) survival analysis (using the “survival” R package) was performed to compare differences in OS. Time-dependent receiver operating characteristic (ROC) curves were also constructed to evaluate the prognostic accuracy of the model. Likewise, we used stepwise Cox linear regression analysis to investigate the influence of clinical parameters on the prognostic signature with the survival package in R. Next, we used stepwise Cox linear PHR analysis to select clinical factors with prognostic characteristics using R programs.

### Statistical Analysis

For all data in our study, prognostic indicators to predict patient survival were filtered out using the corresponding R packages (R version 3.5.2). K–M survival curves with two-sided log-rank test were used to estimate the probability of survival. Differential expression of genes was plotted using GraphPad Prism (version 8.0). Statistical analysis was performed using IBM SPSS 25.0. An independent *t*-test was used to compare differences, with *p* value <0.05 was represented significance.

## Results

### Selection of DNA-Repair-Related Genes in ESCA Patients

**FIGURE 1:**
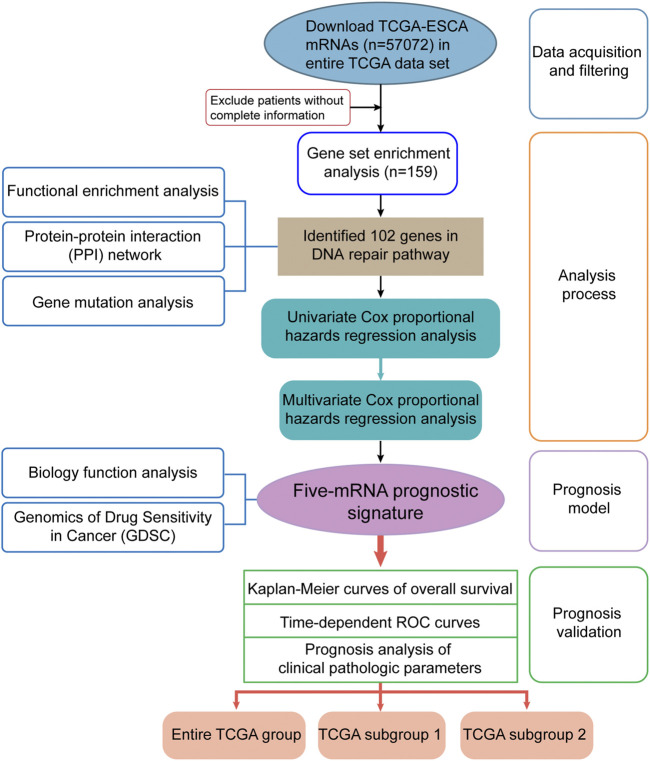
Flow diagram of data and analyses in this work.

**FIGURE 2:**
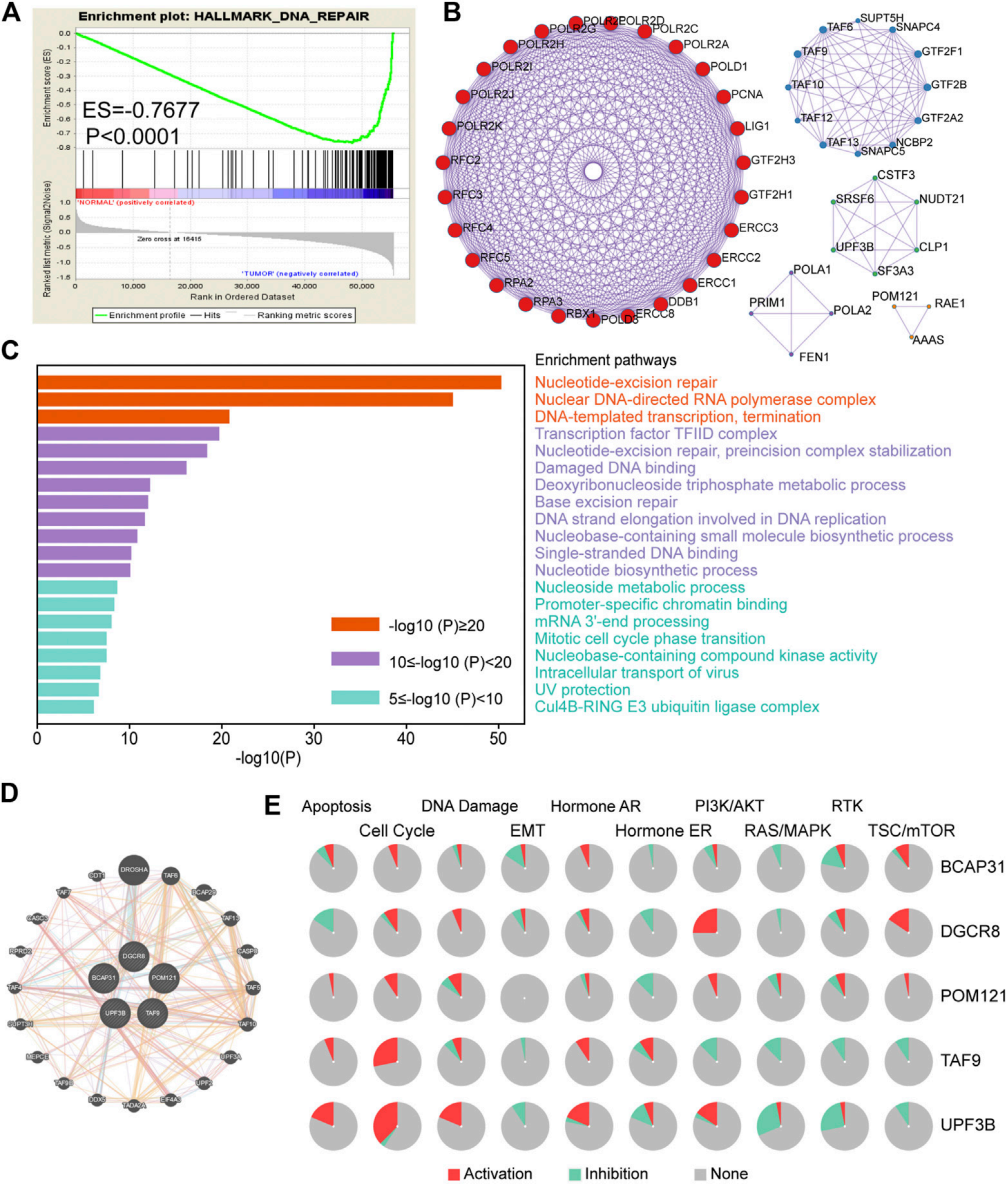
Screening of genes related to DNA repair in ESCA. **(A)** Enrichment plots showing differential expression of DNA-repair-related genes in normal tissues (*n* = 11) and tumor tissues (*n* = 159) according to GSEA. **(B)** Protein–protein interaction network (*n* = 102). **(C)** Functional enrichment (GO and KEGG) analyses of DNA repair genes (*n* = 102). **(D)** Interaction of five genes by GeneMANIA. **(E)** Biological function analysis of the individual five genes in ESCA. Abbreviation: ESCA, esophageal carcinoma; GSEA, gene set enrichment analysis; GO, Gene Ontology; KEGG, Kyoto Encyclopedia of Genes and Genomes.

The detailed workflow of this study is shown in Figure 1. To obtain DNA-repair-related genes, we uploaded 57,072 genes for TCGA-ESCA patients (*n* = 159) to GSEA. Next, we collected 102 genes with *p* < 0.001 that made the greatest contributions to the DNA repair pathway (ESM1: Supplementary Table 1) according to GSEA. The enrichment plot showed that there were statistically significant differences in the identified gene set between the ESCA group and the normal group (Figure 2A). In addition, we analyzed the protein interactions of these genes (Figure 2B, ESM1: Supplementary Table 2). According to the MCODE algorithm, there are three main modes that provide potential value for protein analysis. Biological process enrichment analyses for GO categories and KEGG pathways (Figure 2C) were carried out using the Metascape website. We found that these 102 genes were related to aspects of the DNA repair pathway, including nucleotide-excision repair, DNA-template transcription and termination, damaged DNA binding, base excision repair, nucleotide biosynthetic process, nucleoside metabolic process, and mitotic cell cycle phase transition.

Furthermore, we analyzed the correlation of gene expression with OS based on univariate Cox PHR analysis. As some genes may not have been independent indicators, we applied multivariable Cox PHR analysis to identify the most effective genes. Finally, a five-gene prognostic model comprising DGCR8, POM121, TAF9, UPF3B, and BCAP31 was screened as an independent prognostic biomarker for ESCA patients. We also obtained the hazard ratio (HR, instant probability of reaching alignment) of each gene, as shown in Table 2. For further analysis, we classified these five genes as risk type (HR > 1) or protective type (HR < 1). Therefore, BCAP31, TAF9, and UPF3B were risk-related genes, as their high expression was associated with shorter survival time, whereas DGCR8 and POM121 were protective genes whose high expression meant longer survival time.

**TABLE 2:** The detailed information of selected five genes related to overall survival in patients with ESCA.

Gene	Ensemble ID	B (cox)	HR	*p*-value
BCAP31	ENSG00000185825.14	0.440	1.938	0.0046
TAF9	ENSG00000273841.3	0.397	1.683	0.0243
UPF3B	ENSG00000125351.9	0.383	1.657	0.0048
POM121	ENSG00000196313.10	−0.373	0.603	0.0338
DGCR8	ENSG00000128191.12	−0.864	0.408	0.0070

Abbreviations: ESCA, esophageal cancer; HR, hazard ratio.

In addition, we used GeneMANIA to predict interacting genes and their functions. The results showed that DGCR8 and Drosha (an rnase enzyme) had the strongest correlation (Figure 2D). Notably, both DGCR8 and Drosha have been shown to play important and irreplaceable parts in ultraviolet (UV)-induced DNA damage repair [20]. This also confirmed that the genes we had selected were suitable to construct a robust prognostic model. Besides, pie chart (Figure 2E) was performed to assess the possible mechanisms involving these genes. The results showed that all five genes were related to the cell cycle and DNA damage and could regulate the PI3K/AKT pathway, indicating that they have critical roles in cancer.

### Mutation and Differential Expression Analysis of Five Genes in Signature

**FIGURE 3:**
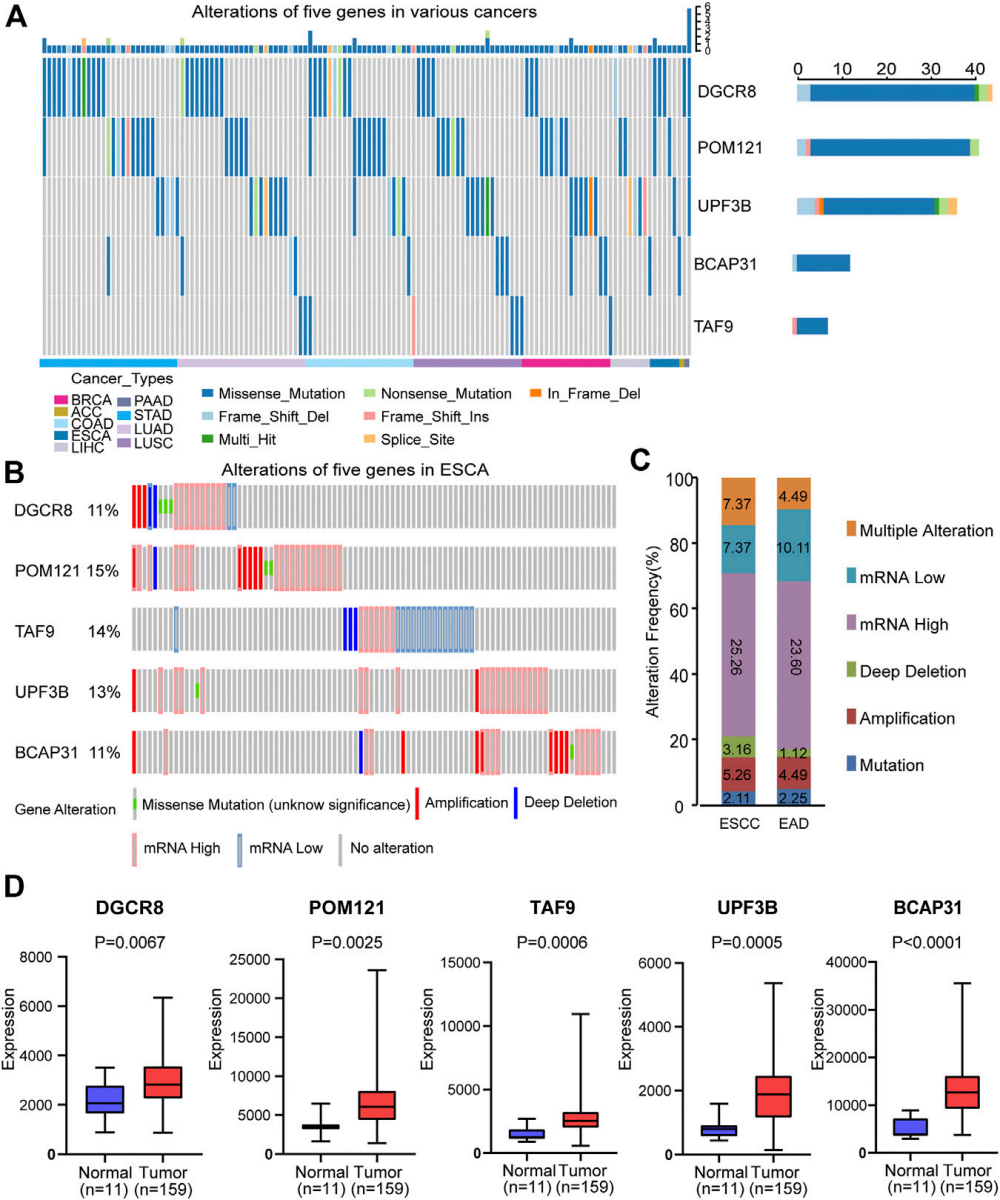
Alterations and differential expression of the five genes. **(A)** Alterations of the five genes in different cancers. **(B)** Alterations of the five genes in ESCA patients. **(C)** Genomic alterations of the five genes in patients with ESCA subtypes. **(D)** Differential expression of the selected five genes in normal group (*n* = 11) and tumor group (*n* = 159). Two-sided log-rank and Wilcoxon *p* < 0.05 were considered significant. Abbreviation: ESCA, esophageal carcinoma; ESCC, esophageal squamous carcinoma; EAD, esophageal adenocarcinoma.

First, we analyzed the alterations of the five genes in different cancers using Metascape. We found that mutations of these genes occurred in various cancers, including ESCA (Figure 3A). Then, we analyzed the changes in the five genes in ESCA samples using the cBioPortal database. For the protective-type genes (DGCR8 and POM121), 11 and 15% of patients showed alterations. For the risk-type genes (UPF3B, BCAP31, and TAF9), 13, 11, and 14% of patients, respectively, showed changes (Figure 3B). These results suggest gene changes may be one research object.

Subsequently, we evaluated the gene alterations in two subtypes including esophageal squamous cell carcinoma (ESCC) and esophageal adenocarcinoma (EAD). Gene alterations in these two subtypes included mutation, amplification, deep deletion, up-regulation, down-regulation and multiple alterations (Figure 3C). The results suggest no significant difference between ESCC and EAD in this regard.

We also compared the expression of the selected five genes in the tumor group (*n* = 159) and the normal group (*n* = 11), and showed that they were significantly up-regulated in tumor tissues (*p* < 0.05, Figure 3D). In addition, in order to further verify that there was significant differential expression of the five genes in the prognostic signature between normal and tumor samples, we performed validation in a independent dataset-GSE38129. As shown in ESM2: Supplementary Figure 1, all five genes were differentially expressed in GSE38129, and the differences were statistically significant (*p* < 0.05).

### Construction of Five-Gene Prognostic Signature

**FIGURE 4:**
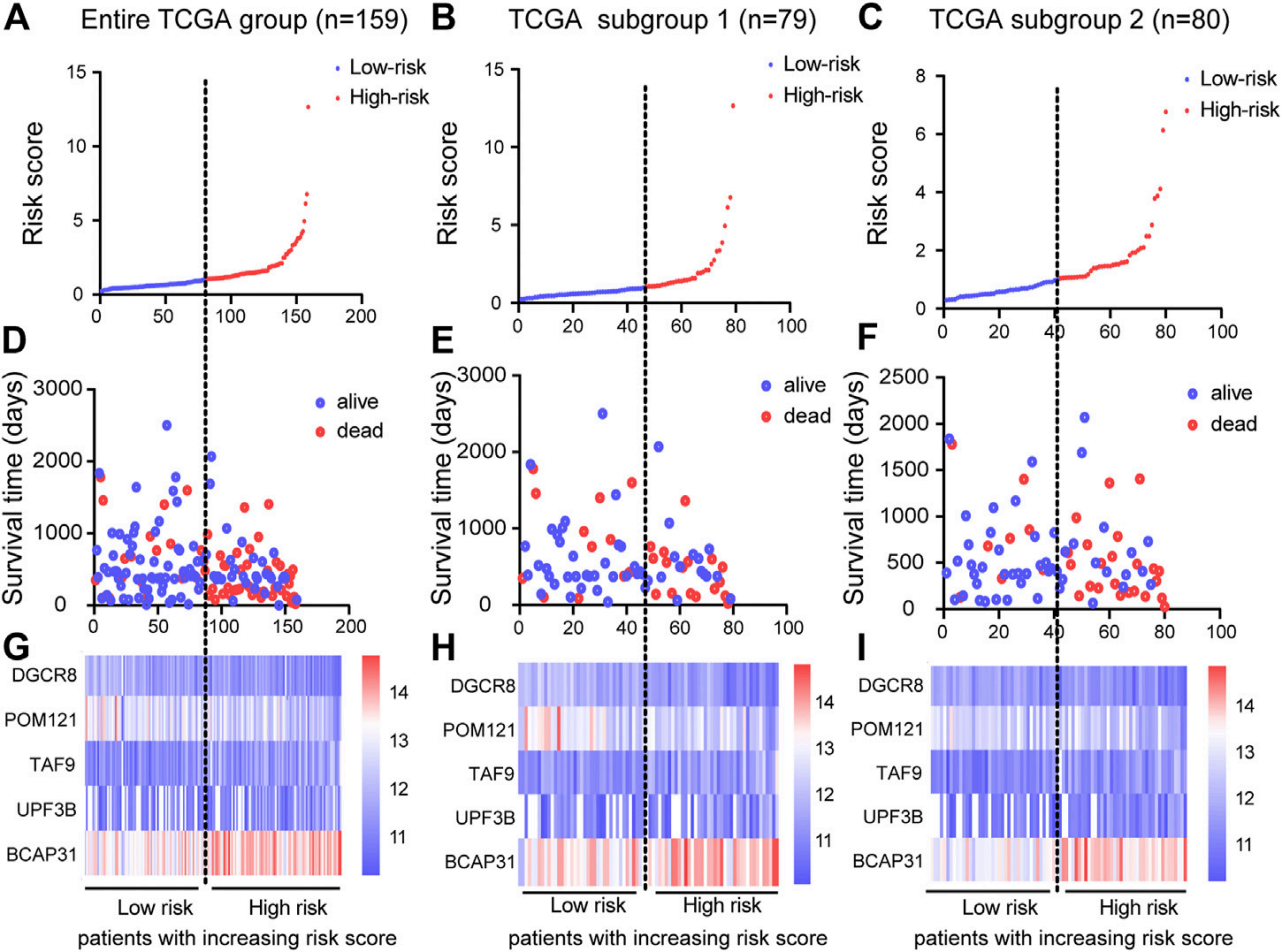
Construction of prognostic risk score system and identification of five-gene prognostic model. Risk score distribution of five genes in three cohorts: entire TCGA group (*n* = 159), TCGA subgroup 1 (*n* = 79), and TCGA subgroup 2 (*n* = 80). Top **(A)–(C)** and middle **(D)–(F)** plots show patient survival time and status based on risk score system **(G)–(I)** Heatmap of expression of the five genes; color from blue to red illustrates a trend from low expression to high expression.

Based on the results of the multivariable Cox PHR analyses, the five genes were used to establish a risk scoring system. We used the risk score formula to calculate a risk score for each patient, and ranked the patients into low- and high-risk groups in the three cohorts according to the median risk score value (Figures 4A–C). We also constructed scatter plots of patient survival time to visualize the survival status of ESCA patients in the three cohorts (Figures 4D–F). Comparison of the two (low and high) risk groups showed that patients with higher risk scores had higher mortality and lower survival rates. In addition, a heatmap (Figures 4G–I) was used to illustrate the expression profile of the five-gene signature. Overall, the results indicate that the risk score had good potential to predict patients’ prognosis.

Next, we analyzed the clinicopathological parameters by stepwise Cox PHR analysis to determine whether the five-gene risk model functioned as an independent prognostic signature when adjusted for cancer stage, stage-M and residual tumor (Table 3). As shown in results, univariate Cox PHR analysis pointed out that five-gene prognostic signature and these clinical pathological factors indeed have prognostic value at the aspect of predicting survival of patients with ESCA. Importantly, five-gene signature, cancer stage and stage M were also independent prognostic indicators with significant differences (*p* < 0.05) both in univariate and multivariate Cox analysis. In particular, risk score had the strongest predictive ability among these indicators (HR 3.388; 95% confidence interval (CI) 1.664–6.899, *p* = 0.001). These results demonstrate that the five-gene signature can effectively predict the prognosis of patients with ESCA and prognostic independent of other clinical factors.

**TABLE 3:** Univariable and multivariable Cox linear regression analysis for risk score and different clinical pathological parameters.

		Univariable analysis			Multivariable analysis		
Clinical feature	Number	HR	95%CI of HR	*p* value	HR	95%CI of HR	*p* value
Risk score (low/high)	79/80	3.819	2.161–6.748	<0.0001	3.388	1.664–6.899	0.001
Cancer stage (stage I-II/III-IV)	87/68	3.182	1.774–5.710	<0.0001	2.732	1.328–5.623	0.006
Stage-M (M0/M1)	126/15	4.92	2.243–10.794	<0.0001	2.535	1.024–6.276	0.044
Residual tumor (R0/R1+ R2)	119/13	2.324	1.143–4.724	0.020	1.199	0.539–2.668	0.657

Abbreviations: HR, hazard ratio; CI, confidence interval.

### Validation of the Prognostic Efficiency of the Five-Gene Signature in Three Cohorts

**FIGURE 5:**
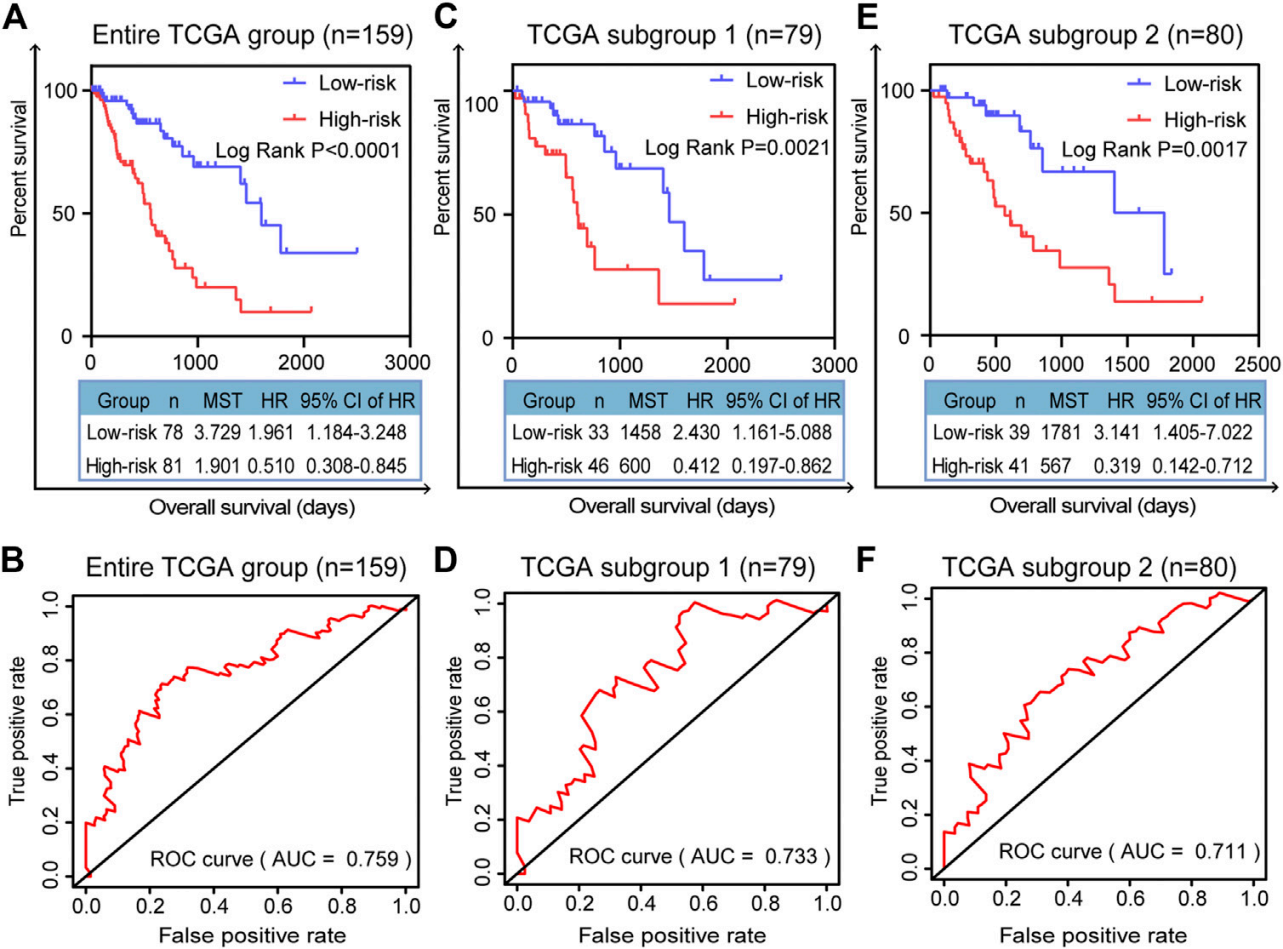
Validation of prognostic signature for patients with ESCA. K–M survival curves for prognostic model and time-dependent ROC curve for **(A, B)** entire TCGA group (*n* = 159), **(C, D)** TCGA subgroup 1 (*n* = 79), and **(E, F)** TCGA subgroup 2 (*n* = 80). Two-sided log-rank and Wilcoxon *p* < 0.05 were considered significant. Abbreviation: MST, median survival time.

We randomly divided all the TCGA-ESCA tumor samples into two subgroups. As well as validation in the entire TCGA group, we validated the prognostic signature using survival curves in these two subgroups. K–M survival curves plotted in the entire TCGA dataset (*n* = 159) showed that the prognostic model stratified patients by OS with significant differences, and the survival rate of high-risk patients was lower than that of low-risk patients (*p* < 0.0001; Figure 5A). The area under the curve (AUC) of the ROC curves showed that the five-gene signature had good predictive performance for ESCA patients (AUC = 0.759; Figure 5B). In the TCGA subgroup 1 (*n* = 79), the K-M survival curve (*p* = 0.0021, Figure 5C) and ROC curve (AUC = 0.733; Figure 5D) also demonstrated that the five-gene model was able to predict the prognosis of ESCA patients. In TCGA subgroup 2 (*n* = 80), the K-M survival curve (*p* = 0.0017, Figure 5E) and ROC curve (AUC = 0.711; Figure 5F) again validated the model. Compared with any of the individual genes (ESM2: Supplementary Figure 2), the five-gene model had better predictive performance as a prognostic indicator in the entire TCGA dataset, with the lowest *p* value (*p* < 0.0001).

### Validation of Independent Prognostic Indicator Under the Influence of Clinical Pathological Factors in Entire TCGA Cohort

**FIGURE 6:**
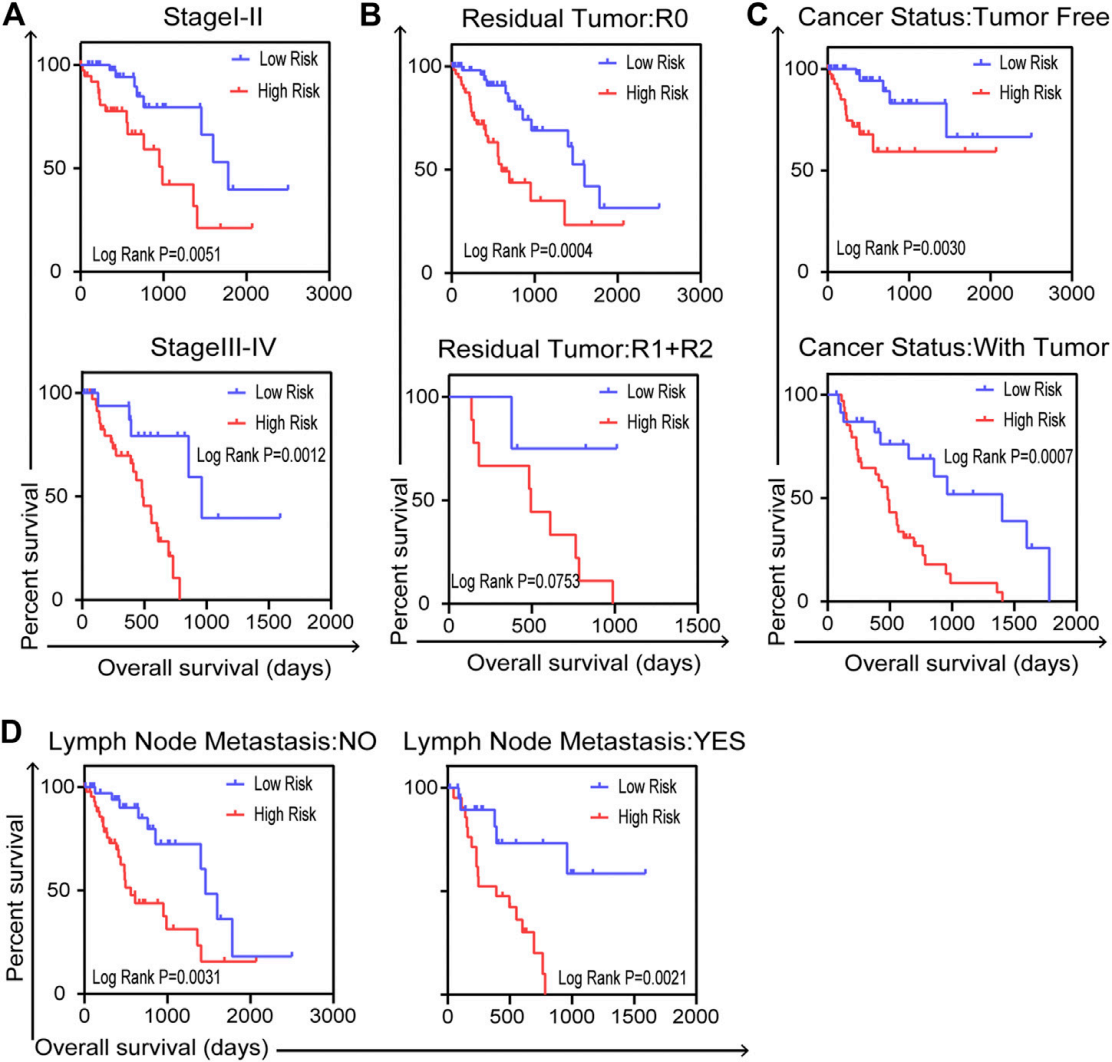
Stratified analysis for further data mining. Validation of the five-gene prognostic signature in patients with ESCA for **(A)** cancer stage, **(B)** residual tumor, **(C)** cancer status and **(D)** lymph node metastasis in entire TCGA dataset (*n* = 159). Two-sided log-rank and Wilcoxon *p* < 0.05 were considered significant.

We carried out further stratified analyses of clinical factors to investigate the clinical value of the prognostic model in the entire TCGA dataset. The results showed that the five-gene signature related to DNA repair was an independent prognostic indicator for patients with ESCA, compared with cancer stage (stage I–II or stage III–IV, Figure 6A), residual tumor status (R0 or R1+R2, Figure 6B), cancer status (tumor free or with tumor, Figure 6C) and lymph node metastasis (no or yes, Figure 6D). But there were no reference values of K–M curves for stage M because of the uneven case numbers of patients. As shown in results, the five-gene signature, as well as having good prognostic value, could serve as an independent prognostic indicator in ESCA patients.

**FIGURE 7:**
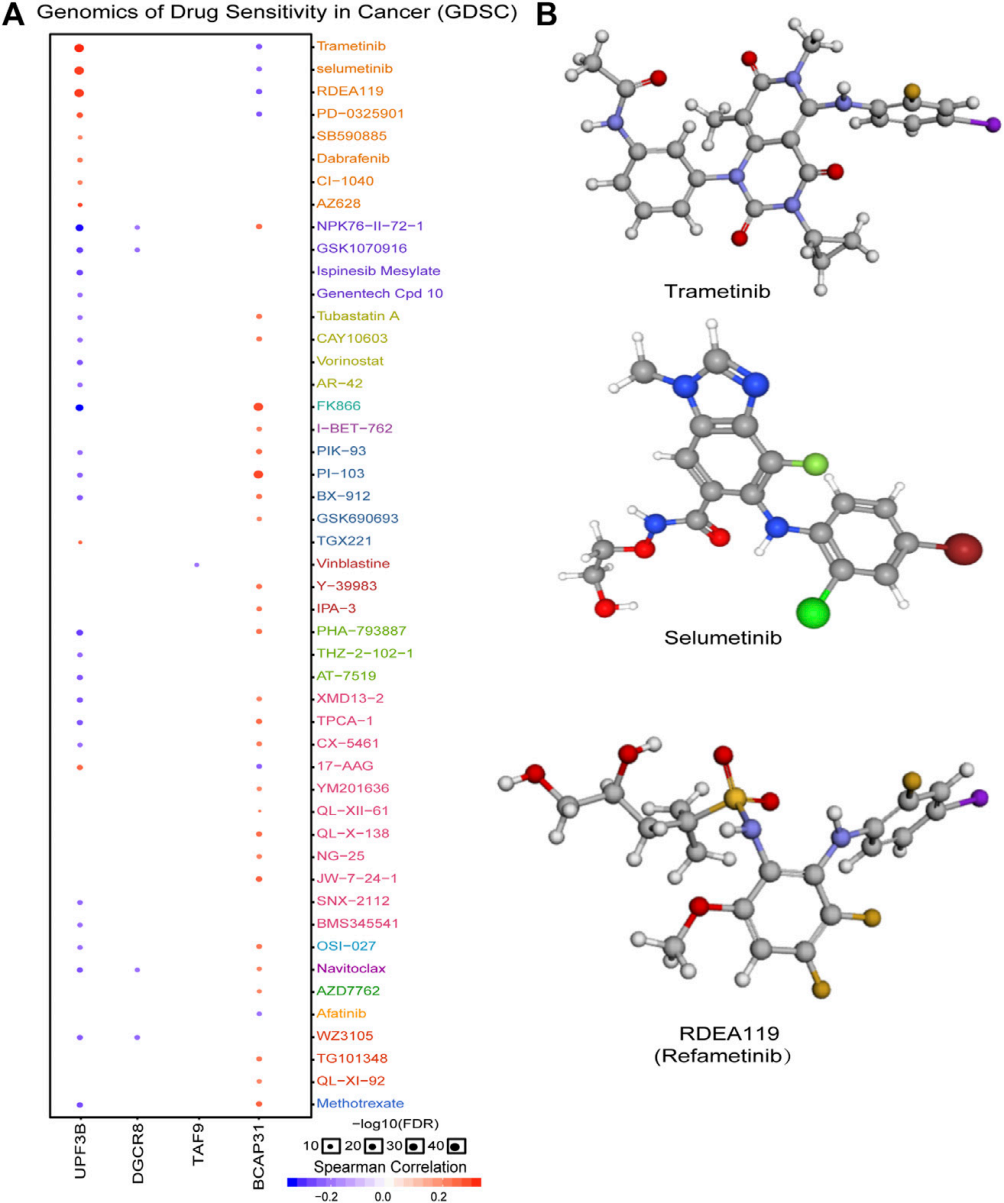
Analysis of potential drug sensitivity of five genes. **(A)** Genomics of drug sensitivity in cancer (GDSC). **(B)** Structure of potential targeted drugs including trametinib, selumetinib and refametinib.

In order to explore molecules that could serve as targeted drugs, we analyzed the drug sensitivity of the five genes in the prognostic signature. As shown in Figure 7A, UPF3B and BCAP31 are more sensitive to drugs. Potential targeted drugs were identified, including trametinib, selumetinib, and refametinib, which could be used to improve patient survival. Based on Spearman correlation analysis, we determined the top three drugs (Figure 7B) with potential for further clinical research.

## Discussion

ESCA is one of the most aggressive cancers, with overall mortality as high as 88% [21]. Although advances in therapeutics have improved clinical outcomes to some extent, the survival rate remains poor. Many biomarkers have been found to be related to survival, and accumulating evidence indicates that gene biomarkers are the preferred way to predict prognosis. Therefore, there is an urgent need to investigate the gene expression profile of ESCA, in order to be able to better assess the prognosis of ESCA patients. Establishing and validating prognostic gene biomarkers may improve clinical outcomes for these patients in the near future.

Recent studies have identified various single genes as biomarkers to reveal the relationship of patients’ survival and cancer progression. For example, DLEU2 [22], FAM60A [23] and CENPE [24] were demonstrated to be independent biomarkers of unfavorable OS in ESCA patients. However, compared with combined markers, single biomarkers are insufficient to independently predict patient prognosis, which can be affected by various factors. Therefore, the application of combined markers in cancer has been reported in succession. For example, a signature of seven long non-coding RNAs (lncRNAs) could indicate survival in ESCC [25]. Integrated analysis led to identification of a three-gene model as a potential biomarker for ESCC [26]. Men and colleagues constructed an 11-gene signature based on the TCGA database that could predict the OS of patients with ovarian cancer [27]. In breast cancer, a five-lncRNA signature has been identified as a prognostic biomarker [28]. Moreover, a prognostic signature including nine genes was shown to have good performance in predicting OS of colorectal cancer patients [29]. Therefore, multi-gene prognostic signatures are necessary for determining cancer prognosis.

DNA damage readily occurs during the cell cycle; it can disturb the cell’s steady state and lead to mutations, cell death, and cancer [30]. In about half of cases, doxorubicin, cisplatin [31] and other chemotherapy drugs will cause huge damage to the DNA of normal cells as well as that of tumor cells during treatment, leading to a limited curative effect and poor prognosis. Notably, DNA repair, DNA damage detection point, transcription reaction and apoptosis are four ways to resume DNA damage. Defects in any of these pathways can lead to genomic instability and cancer. Therefore, DNA damage repair pathways must be considered in future cancer research. Gene markers related to these pathways may play an important part in prediction of patient survival and formulation of cancer treatment strategies. The single genes CD59 [9], RAP80 [10] and SOX17 [11] have been reported to serve as DNA-repair-related biomarkers to predict patients’ prognosis in ESCA or subtypes of this cancer. However, such single-gene signatures are insufficient to predict prognosis. Therefore, we aimed to discover a multi-gene signature related to DNA repair for predicting the survival of ESCA patients.

In this study, through a comprehensive analysis, we developed a DNA-repair-related gene marker to predict the prognosis of patients with ESCA. The vast datasets of TCGA provide an opportunity to systematically analyze mRNA expression profiles in cancer. Therefore, we downloaded mRNA expression profiles for the TCGA-ESCA dataset to find markers that could predict patients’ prognosis. We applied GSEA to identify DNA-repair-related mRNAs, which were subjected to univariate and multivariate Cox PHR analysis. In this way, we obtained a five-gene signature (DGCR8, POM121, TAF9, UPF3B, and BCAP31) as a novel prognostic model. Afterward, according to the Cox coefficient and gene expression values for each patient, a risk scoring system was established in the entire TCGA dataset. Then, we validated the prognostic model using K-M survival curves. The results showed that high-risk patients had a poorer survival rate compared with low-risk patients in the entire TCGA group and in the two subgroups. The AUC of the ROC curve for the five-gene signature was greater than 0.7 in these three cohorts, indicating the strong prognostic value of the signature. Subsequently, validation using clinical factors further indicated that the five-gene signature is an independent indicator in ESCA.

Notably, among the five genes, DGCR8 has been reported to have a critical role in DNA damage response and DNA repair. Studies have shown that DGCR8 together with Drosha (an rnase enzyme) can mediate the repair of UV-induced DNA lesions. Moreover, Swahari and colleagues found that deletion of DGCR8 resulted in DNA damage in the developing mouse brain [32]. DGCR8 is also associated with susceptibility to various cancers [33], including prostate cancer, Wilms tumor, and ovarian cancer. POM121 has been reported to be a key contributor to prostate cancer aggressiveness [34]. In addition, Guo et al [35] found that HIV-1 replication was significantly decreased by small interfering RNA-mediated POM121 knockdown. TAF9 (TATA-binding protein) is one of several histone folding TAFs that maintain the structural integrity [36]. The p53 tumor suppressor gene modulates the activity of the GLI1 oncogene through interactions with the shared activator TAF9 [37]. UPF3B is part of a multi-protein complex that is involved in mRNA nuclear export and the initiation of nonsense-mediated mRNA decay (NMD). About 11% of human genetic diseases are due to NMD, which produces premature translation termination codons in mRNAs. UPF3B has been identified as a potential treatment for NMD-induced diseases, including cancers [38]. BCAP31 (a member of the Bcl-2 protein family) has a potential function in cancer apoptosis, with a role in the proliferation and apoptosis of keratinocytes in cancers. BCAP31 has been reported to be up-regulated in hepatocellular carcinoma [39]; similarly, in our study, BCAP31 was up-regulated in ESCA patients. Another study found that BCAP31 was related to patient survival in breast cancer [40]. However, the role of genes in ESCA patients should be further evaluated.

The advantages of our prognostic predictor are obvious. First, by multistep Cox PHR analysis, we identified a five-gene signature related to DNA repair and the risk coefficient of each patient, so as to build a risk score equation for ESCA patients to be recruited. Next, patients were assigned into two groups by the median risk value according to the equation. Based on the validation results for the clinical pathological parameters, we confirmed that the five-gene signature could effectively predict the prognosis of patients under the influence of different clinical characteristics. This suggests it could predict patients’ prognosis without considering other pathological parameters. The drug sensitivity analysis indicated that small-molecule drugs have potential clinical value for improving patients’ survival outcomes. Although further investigation and experimentation are needed to elucidate the biological mechanisms of the five-gene signature in ESCA development and progression, the prognostic value of the gene signature is promising.

## Conclusion

In conclusion, we identified a novel five-gene predictive model comprising DGCR8, POM121, TAF9, UPF3B, and BCAP31 to indicate prognosis of patients based on integrated bioinformatics analysis. Our study explored the potential clinical significance of this biomarker. The results of the high-throughput data mining show that our prognostic model could independently predict ESCA patients’ survival. These results also provide a theoretical basis for further exploring the molecular pathogenesis of ESCA and identifying therapeutic targets.

## Data Availability

The original contributions presented in the study are included in the article/Supplementary Material, further inquiries can be directed to the corresponding authors.
