# Supplementary material for: Identification of DNA-Repair-Related Five-Gene Signature to Predict Prognosis in Patients with Esophageal Cancer
Source: Pathol Oncol Res. 2021 Mar 30;27:596899. doi: 10.3389/pore.2021.596899 (PMC8262199; doi:10.3389/pore.2021.596899)
Supplement: Supplementary file 1 [file DataSheet1.docx]

**Identification of DNA repair-related five-gene signature to predict the prognosis of patients with esophageal cancer**

**Journal: Pathology & Oncology Research**

Lin Wang^1,2^, Xueping Li^1,2^, Lan Zhao^1,2^, Longyang Jiang^1,2^, Xinyue Song^1,2^, Aoshuang Qi^1,2^, Ting Chen^1,2^, Mingyi Ju^1,2^, Baohui Hu^1,2^, Minjie Wei^1,2^, Miao He^1,2^*, Lin Zhao^1,2^*

^1^ Department of Pharmacology, School of Pharmacy, China Medical University, Shenyang, Liaoning Province, China

^2^ Liaoning Key Laboratory of Molecular Targeted Anti-tumor Drug Development and Evaluation; Liaoning Cancer immune peptide drug Engineering Technology Research Center; Key Laboratory of Precision Diagnosis and Treatment of Gastrointestinal Tumors, Ministry of Education; China Medical University, Shenyang, Liaoning Province, China.

***Corresponding authors:**

Miao He, PhD, E-mail address: [hemiao_cmu@126.com](mailto:hemiao_cmu@126.com).

Lin Zhao, PhD, E-mail address: lzhao@cmu.edu.cn.

**Supplementary Table 1** The information of 102 DNA repair-related genes (*P*<0.001) which were used by stepwise Cox regression analysis in ESCA

| **Gene** | **Ensemble** | **Location** |
| --- | --- | --- |
| DGCR8 | [ENSG00000128191](http://www.ensembl.org/Homo_sapiens/Gene/Summary?g=ENSG00000128191) | [chr22:20080232-20111877](http://genome.ucsc.edu/cgi-bin/hgTracks?db=hg38&position=chr22:20080232-20111877) |
| SEC61A1 | [ENSG00000058262](http://www.ensembl.org/Homo_sapiens/Gene/Summary?g=ENSG00000058262) | [chr3:128051641-128071683](http://genome.ucsc.edu/cgi-bin/hgTracks?db=hg38&position=chr3:128051641-128071683) |
| DDB1 | [ENSG00000167986](http://www.ensembl.org/Homo_sapiens/Gene/Summary?g=ENSG00000167986) | [chr11:61299451-61342596](http://genome.ucsc.edu/cgi-bin/hgTracks?db=hg38&position=chr11:61299451-61342596) |
| ERCC8 | [ENSG00000049167](http://www.ensembl.org/Homo_sapiens/Gene/Summary?g=ENSG00000049167) | [chr5:60873831-60945073](http://genome.ucsc.edu/cgi-bin/hgTracks?db=hg38&position=chr5:60873831-60945073) |
| GUK1 | [ENSG00000143774](http://www.ensembl.org/Homo_sapiens/Gene/Summary?g=ENSG00000143774) | [chr1:228139962-228148984](http://genome.ucsc.edu/cgi-bin/hgTracks?db=hg38&position=chr1:228139962-228148984) |
| ERCC2 | [ENSG00000104884](http://www.ensembl.org/Homo_sapiens/Gene/Summary?g=ENSG00000104884) | [chr19:45349837-45370918](http://genome.ucsc.edu/cgi-bin/hgTracks?db=hg38&position=chr19:45349837-45370918) |
| RNMT | [ENSG00000101654](http://www.ensembl.org/Homo_sapiens/Gene/Summary?g=ENSG00000101654) | [chr18:13726660-13764558](http://genome.ucsc.edu/cgi-bin/hgTracks?db=hg38&position=chr18:13726660-13764558) |
| RRM2B | [ENSG00000048392](http://www.ensembl.org/Homo_sapiens/Gene/Summary?g=ENSG00000048392) | [chr8:102204502-102239118](http://genome.ucsc.edu/cgi-bin/hgTracks?db=hg38&position=chr8:102204502-102239118) |
| AK1 | [ENSG00000106992](http://www.ensembl.org/Homo_sapiens/Gene/Summary?g=ENSG00000106992) | [chr9:127866486-127877675](http://genome.ucsc.edu/cgi-bin/hgTracks?db=hg38&position=chr9:127866486-127877675) |
| SRSF6 | [ENSG00000124193](http://www.ensembl.org/Homo_sapiens/Gene/Summary?g=ENSG00000124193) | [chr20:43457896-43464243](http://genome.ucsc.edu/cgi-bin/hgTracks?db=hg38&position=chr20:43457896-43464243) |
| GMPR2 | [ENSG00000284752](http://www.ensembl.org/Homo_sapiens/Gene/Summary?g=ENSG00000284752) | [chr14:24232422-24239242](http://genome.ucsc.edu/cgi-bin/hgTracks?db=hg38&position=chr14:24232422-24239242) |
| CMPK2 | [ENSG00000134326](http://www.ensembl.org/Homo_sapiens/Gene/Summary?g=ENSG00000134326) | [chr2:6840570-6866635](http://genome.ucsc.edu/cgi-bin/hgTracks?db=hg38&position=chr2:6840570-6866635) |
| VPS37D | [ENSG00000176428](http://www.ensembl.org/Homo_sapiens/Gene/Summary?g=ENSG00000176428) | [chr7:73667831-73672112](http://genome.ucsc.edu/cgi-bin/hgTracks?db=hg38&position=chr7:73667831-73672112) |
| APRT | [ENSG00000198931](http://www.ensembl.org/Homo_sapiens/Gene/Summary?g=ENSG00000198931) | [chr16:88809339-88811944](http://genome.ucsc.edu/cgi-bin/hgTracks?db=hg38&position=chr16:88809339-88811944) |
| POLR1C | [ENSG00000171453](http://www.ensembl.org/Homo_sapiens/Gene/Summary?g=ENSG00000171453) | [chr6:43509702-43562419](http://genome.ucsc.edu/cgi-bin/hgTracks?db=hg38&position=chr6:43509702-43562419) |
| POLD3 | [ENSG00000077514](http://www.ensembl.org/Homo_sapiens/Gene/Summary?g=ENSG00000077514) | [chr11:74493851-74669117](http://genome.ucsc.edu/cgi-bin/hgTracks?db=hg38&position=chr11:74493851-74669117) |
| MPG | [ENSG00000103152](http://www.ensembl.org/Homo_sapiens/Gene/Summary?g=ENSG00000103152) | [chr16:77007-85853](http://genome.ucsc.edu/cgi-bin/hgTracks?db=hg38&position=chr16:77007-85853) |
| POLR1D | [ENSG00000186184](http://www.ensembl.org/Homo_sapiens/Gene/Summary?g=ENSG00000186184) | [chr13:27620742-27744237](http://genome.ucsc.edu/cgi-bin/hgTracks?db=hg38&position=chr13:27620742-27744237) |
| TP53 | [ENSG00000141510](http://www.ensembl.org/Homo_sapiens/Gene/Summary?g=ENSG00000141510) | [chr17:7661779-7687550](http://genome.ucsc.edu/cgi-bin/hgTracks?db=hg38&position=chr17:7661779-7687550) |
| ERCC1 | [ENSG00000012061](http://www.ensembl.org/Homo_sapiens/Gene/Summary?g=ENSG00000012061) | [chr19:45407333-45478828](http://genome.ucsc.edu/cgi-bin/hgTracks?db=hg38&position=chr19:45407333-45478828) |
| VPS28 | [ENSG00000160948](http://www.ensembl.org/Homo_sapiens/Gene/Summary?g=ENSG00000160948) | [chr8:144423601-144428563](http://genome.ucsc.edu/cgi-bin/hgTracks?db=hg38&position=chr8:144423601-144428563) |
| COX17 | [ENSG00000138495](http://www.ensembl.org/Homo_sapiens/Gene/Summary?g=ENSG00000138495) | [chr3:119654513-119677454](http://genome.ucsc.edu/cgi-bin/hgTracks?db=hg38&position=chr3:119654513-119677454) |
| TAF1C | [ENSG00000103168](http://www.ensembl.org/Homo_sapiens/Gene/Summary?g=ENSG00000103168) | [chr16:84177847-84187070](http://genome.ucsc.edu/cgi-bin/hgTracks?db=hg38&position=chr16:84177847-84187070) |
| SF3A3 | [ENSG00000183431](http://www.ensembl.org/Homo_sapiens/Gene/Summary?g=ENSG00000183431) | [chr1:37956975-37990075](http://genome.ucsc.edu/cgi-bin/hgTracks?db=hg38&position=chr1:37956975-37990075) |
| POLA1 | [ENSG00000101868](http://www.ensembl.org/Homo_sapiens/Gene/Summary?g=ENSG00000101868) | [chrX:24693919-24996986](http://genome.ucsc.edu/cgi-bin/hgTracks?db=hg38&position=chrX:24693919-24996986) |
| PNP | [ENSG00000198805](http://www.ensembl.org/Homo_sapiens/Gene/Summary?g=ENSG00000198805) | [chr14:20468954-20477094](http://genome.ucsc.edu/cgi-bin/hgTracks?db=hg38&position=chr14:20468954-20477094) |
| TSG101 | [ENSG00000074319](http://www.ensembl.org/Homo_sapiens/Gene/Summary?g=ENSG00000074319) | [chr11:18468336-18526951](http://genome.ucsc.edu/cgi-bin/hgTracks?db=hg38&position=chr11:18468336-18526951) |
| POLR2A | [ENSG00000284832](http://www.ensembl.org/Homo_sapiens/Gene/Summary?g=ENSG00000284832) | [chr17:7484366-7514616](http://genome.ucsc.edu/cgi-bin/hgTracks?db=hg38&position=chr17:7484366-7514616) |
| TAF13 | [ENSG00000197780](http://www.ensembl.org/Homo_sapiens/Gene/Summary?g=ENSG00000197780) | [chr1:109062486-109076002](http://genome.ucsc.edu/cgi-bin/hgTracks?db=hg38&position=chr1:109062486-109076002) |
| SNAPC5 | [ENSG00000174446](http://www.ensembl.org/Homo_sapiens/Gene/Summary?g=ENSG00000174446) | [chr15:66490135-66497780](http://genome.ucsc.edu/cgi-bin/hgTracks?db=hg38&position=chr15:66490135-66497780) |
| POLR2E | [ENSG00000099817](http://www.ensembl.org/Homo_sapiens/Gene/Summary?g=ENSG00000099817) | [chr19:1086574-1095380](http://genome.ucsc.edu/cgi-bin/hgTracks?db=hg38&position=chr19:1086574-1095380) |
| MRPL40 | [ENSG00000185608](http://www.ensembl.org/Homo_sapiens/Gene/Summary?g=ENSG00000185608) | [chr22:19431902-19436075](http://genome.ucsc.edu/cgi-bin/hgTracks?db=hg38&position=chr22:19431902-19436075) |
| NT5C | [ENSG00000125458](http://www.ensembl.org/Homo_sapiens/Gene/Summary?g=ENSG00000125458) | [chr17:75130225-75131757](http://genome.ucsc.edu/cgi-bin/hgTracks?db=hg38&position=chr17:75130225-75131757) |
| POM121 | [ENSG00000196313](http://www.ensembl.org/Homo_sapiens/Gene/Summary?g=ENSG00000196313) | [chr7:72879365-72951440](http://genome.ucsc.edu/cgi-bin/hgTracks?db=hg38&position=chr7:72879365-72951440) |
| POLR3C | [ENSG00000186141](http://www.ensembl.org/Homo_sapiens/Gene/Summary?g=ENSG00000186141) | [chr1:145824088-145844402](http://genome.ucsc.edu/cgi-bin/hgTracks?db=hg38&position=chr1:145824088-145844402) |
| RALA | [ENSG00000006451](http://www.ensembl.org/Homo_sapiens/Gene/Summary?g=ENSG00000006451) | [chr7:39623565-39708120](http://genome.ucsc.edu/cgi-bin/hgTracks?db=hg38&position=chr7:39623565-39708120) |
| ARL6IP1 | [ENSG00000170540](http://www.ensembl.org/Homo_sapiens/Gene/Summary?g=ENSG00000170540) | [chr16:18791669-18801572](http://genome.ucsc.edu/cgi-bin/hgTracks?db=hg38&position=chr16:18791669-18801572) |
| POLR2D | [ENSG00000144231](http://www.ensembl.org/Homo_sapiens/Gene/Summary?g=ENSG00000144231) | [chr2:127843553-127858155](http://genome.ucsc.edu/cgi-bin/hgTracks?db=hg38&position=chr2:127843553-127858155) |
| UMPS | [ENSG00000114491](http://www.ensembl.org/Homo_sapiens/Gene/Summary?g=ENSG00000114491) | [chr3:124730433-124749273](http://genome.ucsc.edu/cgi-bin/hgTracks?db=hg38&position=chr3:124730433-124749273) |
| GTF2H1 | [ENSG00000110768](http://www.ensembl.org/Homo_sapiens/Gene/Summary?g=ENSG00000110768) | [chr11:18322295-18367045](http://genome.ucsc.edu/cgi-bin/hgTracks?db=hg38&position=chr11:18322295-18367045) |
| CLP1 | [ENSG00000172409](http://www.ensembl.org/Homo_sapiens/Gene/Summary?g=ENSG00000172409) | [chr11:57648992-57661865](http://genome.ucsc.edu/cgi-bin/hgTracks?db=hg38&position=chr11:57648992-57661865) |
| TAF12 | [ENSG00000120656](http://www.ensembl.org/Homo_sapiens/Gene/Summary?g=ENSG00000120656) | [chr1:28589323-28643085](http://genome.ucsc.edu/cgi-bin/hgTracks?db=hg38&position=chr1:28589323-28643085) |
| GTF2B | [ENSG00000137947](http://www.ensembl.org/Homo_sapiens/Gene/Summary?g=ENSG00000137947) | [chr1:88852633-88891944](http://genome.ucsc.edu/cgi-bin/hgTracks?db=hg38&position=chr1:88852633-88891944) |
| GPX4 | [ENSG00000167468](http://www.ensembl.org/Homo_sapiens/Gene/Summary?g=ENSG00000167468) | [chr19:1103926-1106791](http://genome.ucsc.edu/cgi-bin/hgTracks?db=hg38&position=chr19:1103926-1106791) |
| NUDT21 | [ENSG00000167005](http://www.ensembl.org/Homo_sapiens/Gene/Summary?g=ENSG00000167005) | [chr16:56429133-56452199](http://genome.ucsc.edu/cgi-bin/hgTracks?db=hg38&position=chr16:56429133-56452199) |
| RPA2 | [ENSG00000117748](http://www.ensembl.org/Homo_sapiens/Gene/Summary?g=ENSG00000117748) | [chr1:27891524-27914746](http://genome.ucsc.edu/cgi-bin/hgTracks?db=hg38&position=chr1:27891524-27914746) |
| CETN2 | [ENSG00000147400](http://www.ensembl.org/Homo_sapiens/Gene/Summary?g=ENSG00000147400) | [chrX:152826973-152830777](http://genome.ucsc.edu/cgi-bin/hgTracks?db=hg38&position=chrX:152826973-152830777) |
| TAF10 | [ENSG00000166337](http://www.ensembl.org/Homo_sapiens/Gene/Summary?g=ENSG00000166337) | [chr11:6606294-6612539](http://genome.ucsc.edu/cgi-bin/hgTracks?db=hg38&position=chr11:6606294-6612539) |
| DAD1 | [ENSG00000129562](http://www.ensembl.org/Homo_sapiens/Gene/Summary?g=ENSG00000129562) | [chr14:22564907-22589224](http://genome.ucsc.edu/cgi-bin/hgTracks?db=hg38&position=chr14:22564907-22589224) |
| GTF2F1 | [ENSG00000125651](http://www.ensembl.org/Homo_sapiens/Gene/Summary?g=ENSG00000125651) | [chr19:6379572-6393981](http://genome.ucsc.edu/cgi-bin/hgTracks?db=hg38&position=chr19:6379572-6393981) |
| TAF9 | [ENSG00000273841](http://www.ensembl.org/Homo_sapiens/Gene/Summary?g=ENSG00000273841) | [chr5:69364743-69370013](http://genome.ucsc.edu/cgi-bin/hgTracks?db=hg38&position=chr5:69364743-69370013) |
| DDB2 | [ENSG00000134574](http://www.ensembl.org/Homo_sapiens/Gene/Summary?g=ENSG00000134574) | [chr11:47214465-47239240](http://genome.ucsc.edu/cgi-bin/hgTracks?db=hg38&position=chr11:47214465-47239240) |
| SUPT4H1 | [ENSG00000213246](http://www.ensembl.org/Homo_sapiens/Gene/Summary?g=ENSG00000213246) | [chr17:58345175-58353093](http://genome.ucsc.edu/cgi-bin/hgTracks?db=hg38&position=chr17:58345175-58353093) |
| POLR2I | [ENSG00000105258](http://www.ensembl.org/Homo_sapiens/Gene/Summary?g=ENSG00000105258) | [chr19:36113709-36115213](http://genome.ucsc.edu/cgi-bin/hgTracks?db=hg38&position=chr19:36113709-36115213) |
| POLR2C | [ENSG00000102978](http://www.ensembl.org/Homo_sapiens/Gene/Summary?g=ENSG00000102978) | [chr16:57462660-57472009](http://genome.ucsc.edu/cgi-bin/hgTracks?db=hg38&position=chr16:57462660-57472009) |
| SUPT5H | [ENSG00000196235](http://www.ensembl.org/Homo_sapiens/Gene/Summary?g=ENSG00000196235) | [chr19:39436156-39476670](http://genome.ucsc.edu/cgi-bin/hgTracks?db=hg38&position=chr19:39436156-39476670) |
| ERCC3 | [ENSG00000163161](http://www.ensembl.org/Homo_sapiens/Gene/Summary?g=ENSG00000163161) | [chr2:127257290-127294176](http://genome.ucsc.edu/cgi-bin/hgTracks?db=hg38&position=chr2:127257290-127294176) |
| GTF2A2 | [ENSG00000140307](http://www.ensembl.org/Homo_sapiens/Gene/Summary?g=ENSG00000140307) | [chr15:59638062-59657541](http://genome.ucsc.edu/cgi-bin/hgTracks?db=hg38&position=chr15:59638062-59657541) |
| POLE4 | [ENSG00000115350](http://www.ensembl.org/Homo_sapiens/Gene/Summary?g=ENSG00000115350) | [chr2:74958492-74970128](http://genome.ucsc.edu/cgi-bin/hgTracks?db=hg38&position=chr2:74958492-74970128) |
| POLB | [ENSG00000070501](http://www.ensembl.org/Homo_sapiens/Gene/Summary?g=ENSG00000070501) | [chr8:42338454-42371808](http://genome.ucsc.edu/cgi-bin/hgTracks?db=hg38&position=chr8:42338454-42371808) |
| EDF1 | [ENSG00000107223](http://www.ensembl.org/Homo_sapiens/Gene/Summary?g=ENSG00000107223) | [chr9:136862119-136866308](http://genome.ucsc.edu/cgi-bin/hgTracks?db=hg38&position=chr9:136862119-136866308) |
| ZNF707 | [ENSG00000181135](http://www.ensembl.org/Homo_sapiens/Gene/Summary?g=ENSG00000181135) | [chr8:143684452-143713898](http://genome.ucsc.edu/cgi-bin/hgTracks?db=hg38&position=chr8:143684452-143713898) |
| ZNRD1 | [ENSG00000224859](http://www.ensembl.org/Homo_sapiens/Gene/Summary?g=ENSG00000224859) | [chr6:30058899-30064909](http://genome.ucsc.edu/cgi-bin/hgTracks?db=hg38&position=chr6:30058899-30064909) |
| UPF3B | [ENSG00000125351](http://www.ensembl.org/Homo_sapiens/Gene/Summary?g=ENSG00000125351) | [chrX:119805311-119852998](http://genome.ucsc.edu/cgi-bin/hgTracks?db=hg38&position=chrX:119805311-119852998) |
| GTF3C5 | [ENSG00000148308](http://www.ensembl.org/Homo_sapiens/Gene/Summary?g=ENSG00000148308) | [chr9:133030675-133058503](http://genome.ucsc.edu/cgi-bin/hgTracks?db=hg38&position=chr9:133030675-133058503) |
| TMED2 | [ENSG00000086598](http://www.ensembl.org/Homo_sapiens/Gene/Summary?g=ENSG00000086598) | [chr12:123584533-123598582](http://genome.ucsc.edu/cgi-bin/hgTracks?db=hg38&position=chr12:123584533-123598582) |
| SAC3D1 | [ENSG00000168061](http://www.ensembl.org/Homo_sapiens/Gene/Summary?g=ENSG00000168061) | [chr11:65040901-65044828](http://genome.ucsc.edu/cgi-bin/hgTracks?db=hg38&position=chr11:65040901-65044828) |
| SSRP1 | [ENSG00000149136](http://www.ensembl.org/Homo_sapiens/Gene/Summary?g=ENSG00000149136) | [chr11:57325986-57335892](http://genome.ucsc.edu/cgi-bin/hgTracks?db=hg38&position=chr11:57325986-57335892) |
| POLA2 | [ENSG00000014138](http://www.ensembl.org/Homo_sapiens/Gene/Summary?g=ENSG00000014138) | [chr11:65261920-65305589](http://genome.ucsc.edu/cgi-bin/hgTracks?db=hg38&position=chr11:65261920-65305589) |
| SNAPC4 | [ENSG00000165684](http://www.ensembl.org/Homo_sapiens/Gene/Summary?g=ENSG00000165684) | [chr9:136375577-136400168](http://genome.ucsc.edu/cgi-bin/hgTracks?db=hg38&position=chr9:136375577-136400168) |
| ITPA | [ENSG00000125877](http://www.ensembl.org/Homo_sapiens/Gene/Summary?g=ENSG00000125877) | [chr20:3208868-3223870](http://genome.ucsc.edu/cgi-bin/hgTracks?db=hg38&position=chr20:3208868-3223870) |
| ZWINT | [ENSG00000122952](http://www.ensembl.org/Homo_sapiens/Gene/Summary?g=ENSG00000122952) | [chr10:56357227-56361273](http://genome.ucsc.edu/cgi-bin/hgTracks?db=hg38&position=chr10:56357227-56361273) |
| NCBP2 | [ENSG00000114503](http://www.ensembl.org/Homo_sapiens/Gene/Summary?g=ENSG00000114503) | [chr3:196935402-196942594](http://genome.ucsc.edu/cgi-bin/hgTracks?db=hg38&position=chr3:196935402-196942594) |
| TAF6 | [ENSG00000106290](http://www.ensembl.org/Homo_sapiens/Gene/Summary?g=ENSG00000106290) | [chr7:100107070-100119841](http://genome.ucsc.edu/cgi-bin/hgTracks?db=hg38&position=chr7:100107070-100119841) |
| LIG1 | [ENSG00000105486](http://www.ensembl.org/Homo_sapiens/Gene/Summary?g=ENSG00000105486) | [chr19:48115445-48170603](http://genome.ucsc.edu/cgi-bin/hgTracks?db=hg38&position=chr19:48115445-48170603) |
| POLD1 | [ENSG00000062822](http://www.ensembl.org/Homo_sapiens/Gene/Summary?g=ENSG00000062822) | [chr19:50384204-50418016](http://genome.ucsc.edu/cgi-bin/hgTracks?db=hg38&position=chr19:50384204-50418016) |
| RFC3 | [ENSG00000133119](http://www.ensembl.org/Homo_sapiens/Gene/Summary?g=ENSG00000133119) | [chr13:33818069-33966558](http://genome.ucsc.edu/cgi-bin/hgTracks?db=hg38&position=chr13:33818069-33966558) |
| TARBP2 | [ENSG00000139546](http://www.ensembl.org/Homo_sapiens/Gene/Summary?g=ENSG00000139546) | [chr12:53500921-53506431](http://genome.ucsc.edu/cgi-bin/hgTracks?db=hg38&position=chr12:53500921-53506431) |
| GTF2H3 | [ENSG00000111358](http://www.ensembl.org/Homo_sapiens/Gene/Summary?g=ENSG00000111358) | [chr12:123633739-123662604](http://genome.ucsc.edu/cgi-bin/hgTracks?db=hg38&position=chr12:123633739-123662604) |
| AAAS | [ENSG00000094914](http://www.ensembl.org/Homo_sapiens/Gene/Summary?g=ENSG00000094914) | [chr12:53307457-53324864](http://genome.ucsc.edu/cgi-bin/hgTracks?db=hg38&position=chr12:53307457-53324864) |
| POLR2H | [ENSG00000163882](http://www.ensembl.org/Homo_sapiens/Gene/Summary?g=ENSG00000163882) | [chr3:184361718-184368596](http://genome.ucsc.edu/cgi-bin/hgTracks?db=hg38&position=chr3:184361718-184368596) |
| POLR2J | [ENSG00000005075](http://www.ensembl.org/Homo_sapiens/Gene/Summary?g=ENSG00000005075) | [chr7:102473118-102478907](http://genome.ucsc.edu/cgi-bin/hgTracks?db=hg38&position=chr7:102473118-102478907) |
| DUT | [ENSG00000128951](http://www.ensembl.org/Homo_sapiens/Gene/Summary?g=ENSG00000128951) | [chr15:48331011-48343373](http://genome.ucsc.edu/cgi-bin/hgTracks?db=hg38&position=chr15:48331011-48343373) |
| NME1 | [ENSG00000239672](http://www.ensembl.org/Homo_sapiens/Gene/Summary?g=ENSG00000239672) | [chr17:51153536-51162428](http://genome.ucsc.edu/cgi-bin/hgTracks?db=hg38&position=chr17:51153536-51162428) |
| TYMS | [ENSG00000176890](http://www.ensembl.org/Homo_sapiens/Gene/Summary?g=ENSG00000176890) | [chr18:657653-673578](http://genome.ucsc.edu/cgi-bin/hgTracks?db=hg38&position=chr18:657653-673578) |
| FEN1 | [ENSG00000168496](http://www.ensembl.org/Homo_sapiens/Gene/Summary?g=ENSG00000168496) | [chr11:61792911-61797238](http://genome.ucsc.edu/cgi-bin/hgTracks?db=hg38&position=chr11:61792911-61797238) |
| PRIM1 | [ENSG00000198056](http://www.ensembl.org/Homo_sapiens/Gene/Summary?g=ENSG00000198056) | [chr12:56731580-56752373](http://genome.ucsc.edu/cgi-bin/hgTracks?db=hg38&position=chr12:56731580-56752373) |
| RBX1 | [ENSG00000100387](http://www.ensembl.org/Homo_sapiens/Gene/Summary?g=ENSG00000100387) | [chr22:40951347-40973309](http://genome.ucsc.edu/cgi-bin/hgTracks?db=hg38&position=chr22:40951347-40973309) |
| BCAP31 | [ENSG00000185825](http://www.ensembl.org/Homo_sapiens/Gene/Summary?g=ENSG00000185825) | [chrX:153700492-153724746](http://genome.ucsc.edu/cgi-bin/hgTracks?db=hg38&position=chrX:153700492-153724746) |
| RAD51 | [ENSG00000051180](http://www.ensembl.org/Homo_sapiens/Gene/Summary?g=ENSG00000051180) | [chr15:40694774-40732340](http://genome.ucsc.edu/cgi-bin/hgTracks?db=hg38&position=chr15:40694774-40732340) |
| POLR2G | [ENSG00000168002](http://www.ensembl.org/Homo_sapiens/Gene/Summary?g=ENSG00000168002) | [chr11:62761565-62766710](http://genome.ucsc.edu/cgi-bin/hgTracks?db=hg38&position=chr11:62761565-62766710) |
| CSTF3 | [ENSG00000176102](http://www.ensembl.org/Homo_sapiens/Gene/Summary?g=ENSG00000176102) | [chr11:33077188-33162371](http://genome.ucsc.edu/cgi-bin/hgTracks?db=hg38&position=chr11:33077188-33162371) |
| HPRT1 | [ENSG00000165704](http://www.ensembl.org/Homo_sapiens/Gene/Summary?g=ENSG00000165704) | [chrX:134460165-134520513](http://genome.ucsc.edu/cgi-bin/hgTracks?db=hg38&position=chrX:134460165-134520513) |
| POLR2K | [ENSG00000147669](http://www.ensembl.org/Homo_sapiens/Gene/Summary?g=ENSG00000147669) | [chr8:100150623-100154003](http://genome.ucsc.edu/cgi-bin/hgTracks?db=hg38&position=chr8:100150623-100154003) |
| RPA3 | [ENSG00000106399](http://www.ensembl.org/Homo_sapiens/Gene/Summary?g=ENSG00000106399) | [chr7:7636518-7718607](http://genome.ucsc.edu/cgi-bin/hgTracks?db=hg38&position=chr7:7636518-7718607) |
| PCNA | [ENSG00000132646](http://www.ensembl.org/Homo_sapiens/Gene/Summary?g=ENSG00000132646) | [chr20:5114953-5126626](http://genome.ucsc.edu/cgi-bin/hgTracks?db=hg38&position=chr20:5114953-5126626) |
| RFC5 | [ENSG00000111445](http://www.ensembl.org/Homo_sapiens/Gene/Summary?g=ENSG00000111445) | [chr12:118013588-118033130](http://genome.ucsc.edu/cgi-bin/hgTracks?db=hg38&position=chr12:118013588-118033130) |
| RFC4 | [ENSG00000163918](http://www.ensembl.org/Homo_sapiens/Gene/Summary?g=ENSG00000163918) | [chr3:186789880-186807058](http://genome.ucsc.edu/cgi-bin/hgTracks?db=hg38&position=chr3:186789880-186807058) |
| RFC2 | [ENSG00000049541](http://www.ensembl.org/Homo_sapiens/Gene/Summary?g=ENSG00000049541) | [chr7:74231499-74254458](http://genome.ucsc.edu/cgi-bin/hgTracks?db=hg38&position=chr7:74231499-74254458) |
| RAE1 | [ENSG00000101146](http://www.ensembl.org/Homo_sapiens/Gene/Summary?g=ENSG00000101146) | [chr20:57351223-57379211](http://genome.ucsc.edu/cgi-bin/hgTracks?db=hg38&position=chr20:57351223-57379211) |
| ADRM1 | [ENSG00000130706](http://www.ensembl.org/Homo_sapiens/Gene/Summary?g=ENSG00000130706) | [chr20:62302093-62308862](http://genome.ucsc.edu/cgi-bin/hgTracks?db=hg38&position=chr20:62302093-62308862) |
| DGUOK | [ENSG00000114956](http://www.ensembl.org/Homo_sapiens/Gene/Summary?g=ENSG00000114956) | [chr2:73926826-73958961](http://genome.ucsc.edu/cgi-bin/hgTracks?db=hg38&position=chr2:73926826-73958961) |

**Abbreviations:** ESCA, esophageal cancer.

**Supplementary Table 2** The information of protein-protein interaction network

| **MCODE** | **Pathways** | **Description** | **Log10(P)** |
| --- | --- | --- | --- |
| MCODE_1 | GO:0006283 | transcription-coupled nucleotide-excision repair | -70.4 |
| MCODE_1 | GO:0006289 | nucleotide-excision repair | -64.4 |
| MCODE_1 | GO:0006281 | DNA repair | -44.4 |
| MCODE_2 | GO:0042795 | snRNA transcription by RNA polymerase II | -20.7 |
| MCODE_2 | GO:0009301 | snRNA transcription | -20.6 |
| MCODE_2 | GO:0005669 | transcription factor TFIID complex | -20.3 |
| MCODE_3 | GO:0031124 | mRNA 3'-end processing | -11.2 |
| MCODE_3 | GO:0000398 | mRNA splicing, via spliceosome | -11.1 |
| MCODE_3 | GO:0000377 | RNA splicing, via transesterification reactions with bulged adenosine as nucleophile | -11.1 |
| MCODE_4 | GO:0032201 | telomere maintenance via semi-conservative replication | -11.9 |
| MCODE_4 | hsa03030(KEGG) | DNA replication | -11.4 |
| MCODE_4 | hsaM00261(KEGG) | DNA polymerase alpha / primase complex | -11.2 |
| MCODE_5 | GO:0006409 | tRNA export from nucleus | -8.6 |
| MCODE_5 | GO:0071431 | tRNA-containing ribonucleoprotein complex export from nucleus | -8.6 |
| MCODE_5 | GO:0051031 | tRNA transport | -8.5 |

**Abbreviations:** GO, Gene Oncology; KEGG, Kyoto Encyclopedia of Genes and Genomes.
