# Supplementary material for: Identification of DNA-Repair-Related Five-Gene Signature to Predict Prognosis in Patients with Esophageal Cancer
Source: Pathol Oncol Res. 2021 Mar 30;27:596899. doi: 10.3389/pore.2021.596899 (PMC8262199; doi:10.3389/pore.2021.596899)
Supplement: Supplementary file 2 [file DataSheet2.docx]

**Identification of DNA repair-related five-gene signature to predict the prognosis of patients with esophageal cancer**

**Journal: Pathology & Oncology Research**

Lin Wang^1,2^, Xueping Li^1,2^, Lan Zhao^1,2^, Longyang Jiang^1,2^, Xinyue Song^1,2^, Aoshuang Qi^1,2^, Ting Chen^1,2^, Mingyi Ju^1,2^, Baohui Hu^1,2^, Minjie Wei^1,2^, Miao He^1,2^*, Lin Zhao^1,2^*

^1^ Department of Pharmacology, School of Pharmacy, China Medical University, Shenyang, Liaoning Province, China

^2^ Liaoning Key Laboratory of Molecular Targeted Anti-tumor Drug Development and Evaluation; Liaoning Cancer immune peptide drug Engineering Technology Research Center; Key Laboratory of Precision Diagnosis and Treatment of Gastrointestinal Tumors, Ministry of Education; China Medical University, Shenyang, Liaoning Province, China.

***Corresponding authors:**

Miao He, PhD, E-mail address: [hemiao_cmu@126.com](mailto:hemiao_cmu@126.com).

Lin Zhao, PhD, E-mail address: lzhao@cmu.edu.cn.


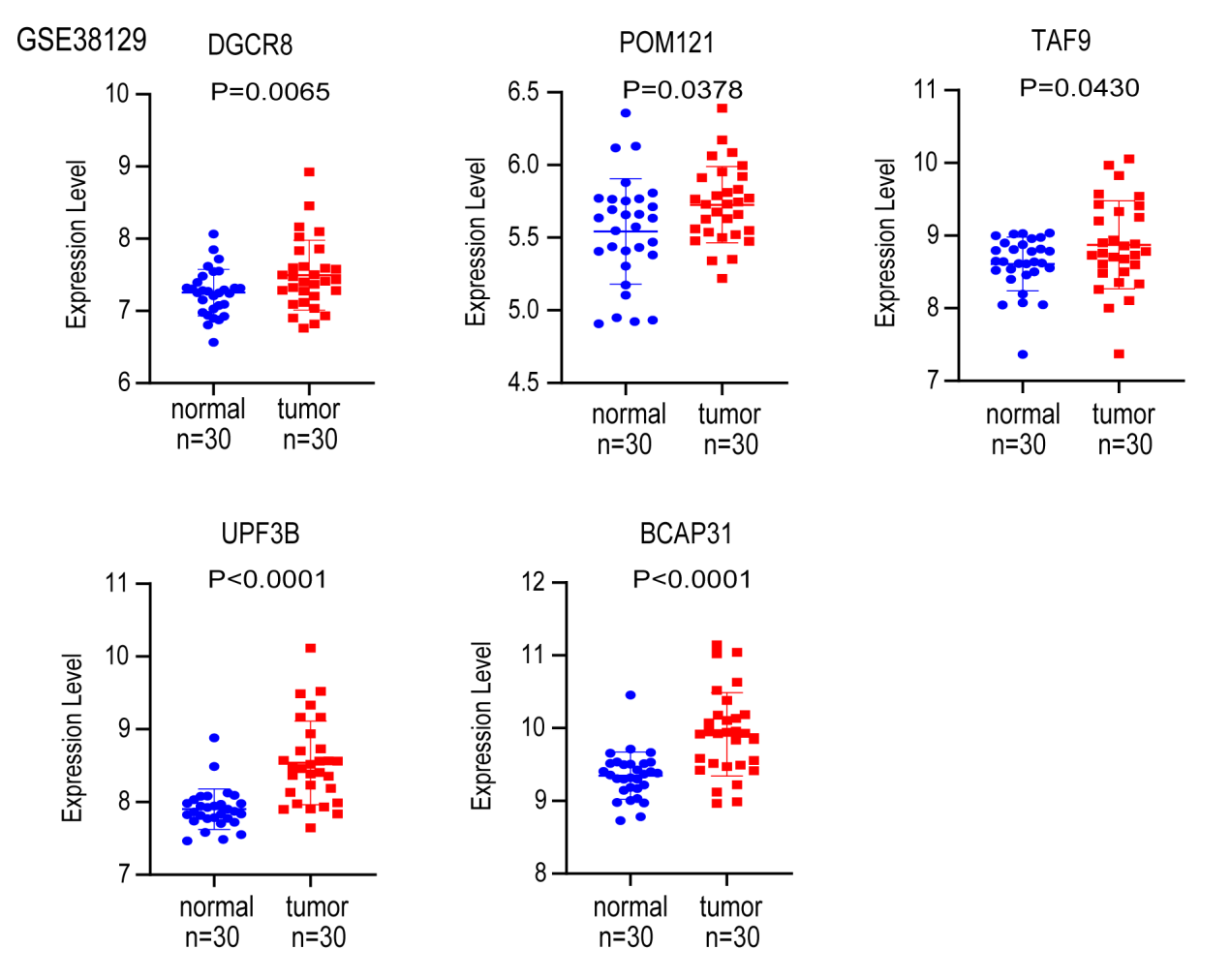


**Supplementary Figure 1 The differential analysis of five genes in prognostic model.** The differential expression of each genes including DGCR8, POM121, TAF9, UPF3B and BPAP31 in GSE38129 (n=60). *P*<0.05 were considered significant.

**
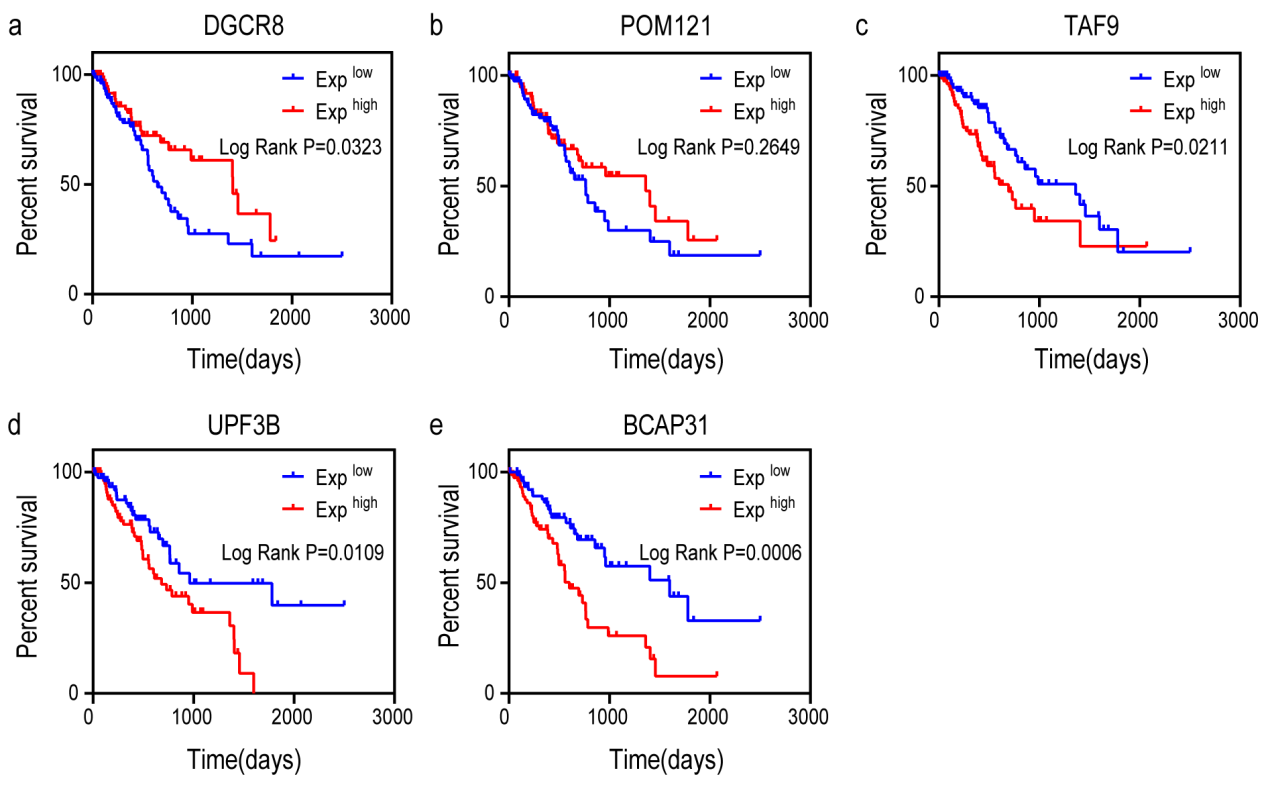
**

**Supplementary Figure 2** **The survival analysis of five genes in ESCA.** The Kaplan-Meier survival curves of each genes including (a) DGCR8, (b) POM121, (c) TAF9, (d) UPF3B and (e) BPAP31 in entire TCGA cohort (n=159). A two-sided Log-Rank and Wilcoxon *P*<0.05 were considered significant.
